# Pilot Scale Application of a Ceramic Membrane Bioreactor for Treating High-Salinity Oil Production Wastewater

**DOI:** 10.3390/membranes12050473

**Published:** 2022-04-27

**Authors:** Ronglin Sun, Yue Jin

**Affiliations:** 1 Guangxi Key Laboratory of Theory & Technology for Environmental Pollution Control, College of Environmental Science and Engineering, Guilin University of Technology, Guilin 541004, China; 2120200447@glut.edu.cn; 2 Guangxi Collaborative Innovation Center for Water Pollution Control and Water Safety in Karst Area, Guilin University of Technology, Guilin 541004, China; 3 College of Civil Engineering and Architecture, Guilin University of Technology, Guilin 541004, China

**Keywords:** high-salinity oil-bearing production wastewater, MBR combination process, functional microorganisms, membrane contamination, economic analysis

## Abstract

The offshore oil extraction process generates copious amounts of high-salinity oil-bearing wastewater; at present, treating such wastewater in an efficient and low-consumption manner is a major challenge. In this study, a flat ceramic membrane bioreactor (C−MBR) process combining aerobic microbial treatment technology and ceramic membrane filtration technology was used to treat oil-bearing wastewater. The pilot test results demonstrated the remarkable performance of the combined sequential batch reactor (SBR) and C-MBR process, wherein the chemical oxygen demand (COD) and ammonia nitrogen (NH_4_^+^−N) removal rates reached 93% and 98.9%, respectively. Microbial analysis indicated that the symbiosis between *Marinobacterium*, *Marinobacter*, and *Nitrosomonas* might have contributed to simultaneously removing NH_4_^+^−N and reducing COD, and the increased enrichment of *Nitrosomonas* significantly improved the nitrogen removal efficiency. Cleaning ceramic membranes with NaClO solution reduces membrane contamination and membrane cleaning frequency. The combined SBR and C−MBR process is an economical and feasible solution for treating high-salinity oil-bearing wastewater. Based on the pilot application study, the capital expenditure for operating the full-scale combined SBR and C−MBR process was estimated to be 251,717 USD/year, and the unit wastewater treatment cost was 0.21 USD/m^3^, which saved 62.5% of the energy cost compared to the conventional MBR process.

## 1. Introduction

The global demand for energy has led to a boom in the oil and gas industry. With the expansion of the extraction scale in each oil field, the volume of wastewater (i.e., produced water, PW) generated during the extraction process is rapidly increasing. Furthermore, PW contains large amounts of hydrocarbons, heavy metals, aromatic compounds, and naturally occurring radioactive substances [1,2]. Discharging such pollutants into the environment without effective treatment not only disturbs the marine ecosystem and affects human health but also hinders oil extraction efforts [3,4,5]. Treating extracted water that contains a high organic load and exhibits poor biochemical properties using biological methods is difficult. Well-established physical and chemical methods, including adsorption technology, membrane filtration technology, and electrochemical methods, have been used to treat PW [6,7,8,9]. However, considering the economics, these physical and chemical methods are expensive, energy-intensive, and operationally complex for large-scale applications. Biological methods, which are inexpensive and environmentally friendly, are more aligned with the concept of sustainable development compared with physical and chemical methods. Biological methods have been successfully used for treating oil recovery wastewater [10,11,12]. Chen et al. [13] applied the combined sequential batch reactor (SBR) process to treat oilfield PW and successfully reduced the chemical oxygen demand (COD) by 88%. Zhang et al. [14] used an anaerobic baffle reactor (ABR) −SBR process for the first time to treat high-salinity wastewater from the oil recovery terminal platform of a the China National Offshore Oil Corporation (CNOOC) and achieved a remarkable COD removal effect.

However, most biological treatment processes are ineffective in removing ammonia nitrogen (NH_4_^+^−N). The NH_4_^+^−N in the effluent continues to exceed the standard value, and the treated water volume remains unstable; these key issues limit the development of biological treatment processes. Excessive NH_4_^+^−N discharge likely causes eutrophication around the drilling platform and damages the marine ecological balance; the loss of microorganisms during process operation is the main cause of this problem. A membrane bioreactor (MBR) combining membrane filtration technology and biological treatment technology can effectively separate activated sludge and wastewater, which effectively enriches and maintains nitrifying bacteria at a certain concentration, thereby enhancing denitrification efficiency. In recent years, MBR technology, with its extensive applications, has observed a rapid growth domestically and internationally and has often been used to treat the main pollution indicators, namely, COD and NH_4_^+^−N, and a few other pollution indicators, to meet the standards for discharge or reuse [15,16,17]. Li et al. [18] used an anaerobic-aerobic (A/O) reflux process to treat high-concentration oily wastewater and observed 93.2% and 82.8% removal rates for COD and TN, respectively, where MBR provided 95% and 99% removal rates, respectively, which demonstrated their remarkable efficiency in treating high-concentration oily wastewater. In addition, MBR processes based on flat ceramic membranes (i.e., C−MBR processes) consume less energy than those based on organic membranes [19]. However, membrane contamination continues to be one of the major problems that hinder the application of C−MBR processes.

The combined use of membrane filtration technology and aerobic activated sludge method for treating oil production wastewater has been studied domestically and internationally, but there exist few relevant examples of its practical application. Considering the above factors, the main objective of this study was to conduct a pilot application study of the C−MBR process for treating high-salinity oil production wastewater to evaluate the removal efficiency, microbial community structure, and membrane contamination. To understand the major aspects of this process, achieve good treatment effects, and apply them in practice, this study analyzed the results by considering COD and NH_4_^+^−N as the main water pollution indexes. Furthermore, we examined the feasibility of the process and summarized the elements that are responsible for the smooth process operation and that help maintain good and stable effluent quality, and we provided an economic analysis of the process.

## 2. Materials and Methods

### 2.1. C−MBR Pilot Plant

**Figure 1:**
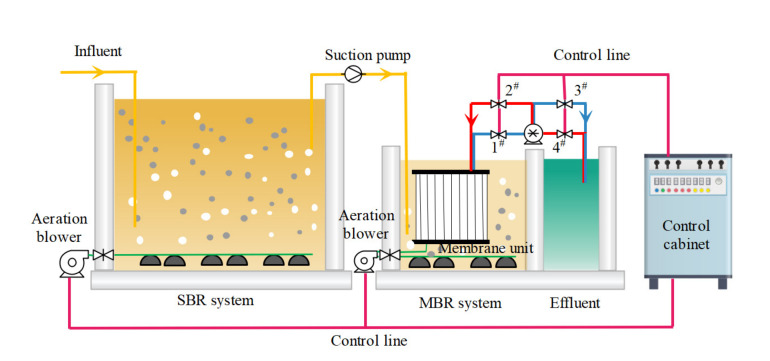
Flat ceramic membrane bioreactor (C-MBR) device for the pilot application. (1# and 3# are filter solenoid valves; 2# and 4# are backwash solenoid valves).

The pilot-scale C−MBR process is shown in Figure 1. The aerobic bioreactor is an open-ended rectangular reactor with dimensions 2620 × 1750 × 600 mm and an effective volume of 2.751 m^3^. The MBR reactor, also a rectangular body with dimensions 1070 × 700 × 600 mm, uses a ceramic membrane supplied by the Meidensha Group of Japan with a pore size of 0.1 μm (particle capture rate of more than 95%). The membrane filtration method followed was internal suction and external filtration, with dimensions of 280 × 1046 × 12 mm, a mass (dry) of 2.2 kg, and an effective membrane area of 0.5 m^2^.

The membrane module is equipped with a backwashing system that utilizes a filtration time of 9 min and a backwashing time of 1 min. When the 1# and 3# solenoid valves are opened, the 2# and 4# solenoid valves are closed, which initiates the sewage filtration process for 9 min; when the 2# and 4# solenoid valves are opened, the 1# and 3# solenoid valves are closed, which initiates the backwashing process of the ceramic flat membrane for 1 min.

### 2.2. Wastewater Composition

The wastewater used in this study was produced from a terminal oil production platform of CNOOC. The main wastewater characteristics include complex components, high salt contents, low transparency, high COD and oil contents, and poor biodegradability (BOD_5_/COD < 0.03). The highest value for COD in the influent measured 856.8 mg/L, and the nitrogen volume load (NLR) was 0.01 g/m^3^·d. The main wastewater quality parameters are shown in Table 1 [14].

### 2.3. Reactor Operation Process

The entire test process was divided into two operation stages. The aerobic activated sludge used in this study was taken from the existing aerobic biological treatment tank in the company’s factory area. It was acclimated for a prolonged period in the oil production wastewater; thus, the water inflow test was performed directly. During the start of the reactor, the amount of sludge added to the SBR and MBR was one-third and two-thirds of the reactor volume, respectively.

The first stage operation process was operated for a total of 19 d. The hydraulic retention time (HRT) of the C−MBR reactor was set to 3 d. A separate C−MBR reactor, which is the right half of the entire reaction system (Figure 1), was used. A certain amount of production wastewater was used as feed water every day, and raw water was pumped into the reactor using the feed pump and then mixed with the activated sludge in the aerated reactor. The effluent and sludge mixture was efficiently separated using the membrane module. The treated effluent was pumped by the filter pump into the discharge tank and released. The membrane module was backwashed periodically by the control cabinet and solenoid valve, as well as the scouring effect of the water bubbles on the membrane during aeration via the aeration tube, to clean the contaminants on the surface ceramic flat membrane and reduce membrane module blockages. The pumping and aeration time ratio was 9/1.

The second stage operation process was operated for 8 d. To increase the daily wastewater treatment capacity and achieve a better treatment effect, SBR with a maximum volume of 2.751 m^3^ was attached to the reaction system in the first step. The daily treatment capacity of MBR was increased from 20 L to 200 L, and the HRT was gradually adjusted from 16.8 h to 4.8 h. A certain amount of wastewater was added directly into the SBR for aeration treatment every day, after which the supernatant was transferred into the inlet bucket after 23 h of aeration and 1 h of settling. The supernatant was pumped into the MBR for further treatment. After the aerobic activated sludge treatment and membrane separation in the MBR, the treated effluent was filtered by the discharge pump into the discharge tank and released. In the two stages, the MBR always maintained 24 h of uninterrupted aeration and continuous discharge, whereas the SBR maintained 23 h of aeration and 1 h of sedimentation per day.

During the operation, COD, NH_4_^+^−N, pH, temperature, transmembrane pressure (TMP), and other indicators of the reactor influent and effluent were measured and recorded via daily sampling.

### 2.4. Analysis Method

#### 2.4.1. Water Quality Analysis

COD was measured using the rapid sealed microwave digestion method (WMX-III-B microwave sealed digestion COD speed tester); NH_4_^+^−N was detected using a Thermo Scientific 2240 ammonia nitrogen online tester (Thermo Fisher Scientific, Shanghai, China); pH and temperature were measured using an PHB-4 portable pH thermometer (LEICI, Shanghai, China); dissolved oxygen (DO) was measured using the Shanghai Remag JPB-607, which is a portable DO analysis tester; and total salinity was measured via the weight method of calculation. TMP was recorded using a digital pressure sensor (Shangyi; Foshan, China). The membrane surfaces were observed by scanning the ceramic membranes before and after cleaning with a field emission scanning electron microscope (s−4800, Hitachi, Tokyo, Japan).

#### 2.4.2. Microbial Diversity Analysis

Aerobic activated sludge sample A_0_ from a common wastewater treatment plant and sludge sample B cultured in the MBR system were collected for microbial diversity testing. The samples were dewatered using a centrifuge and stored in a refrigerator at −20 °C. The microbial diversity assay process included the following: (1) sample DNA extraction, (2) sample DNA integrity detection using agarose gel, (3) polymerase chain reaction (PCR) amplification, (4) DNA purification and recovery, and (5) quantitative mixing for sequencing. The specific experimental steps were as follows. First, DNA was extracted from sludge samples using a PowerSoil DNA isolation kit (Mo Bio, Carlsbad, CA, USA) according to the manufacturer’s instructions. The purity of the extracted DNA was tested (OD260/OD280 of 1.6–1.8), and then it was amplified using PCR. The target fragment amplification and target DNA fragment cleavage were performed simultaneously using PCR. The target DNA fragments were separated and purified via denaturing gradient gel electrophoresis (DGGE), wherein DNA fragments of different microorganisms were immobilized at different positions in the gel. After each gelation step, a band was recovered and re-solubilized to purify the target DNA fragments. The PCR samples were sequenced by Shanghai Biotech Biological Co., Ltd. (Shanghai, China), and the sequenced genes were compared with those stored in the NCBI GenBank database using BLAST analysis.

#### 2.4.3. Data Analysis

Origin 2022 software was used to prepare comparative plots of COD, NH_4_^+^−N, and SS removal rates for different processes, including the single C−MBR process in the first stage and the combined SBR and C−MBR process in the second stage. Correlation analysis of the C/N ratio, COD levels, and NH_4_^+^−N levels and their removal were performed using IBM SPSS Statistics 22 software.

## 3. Results and Discussion

### 3.1. System Processing Performance

#### 3.1.1. Chemical Oxygen Demand (COD) Removal

**Figure 2:**
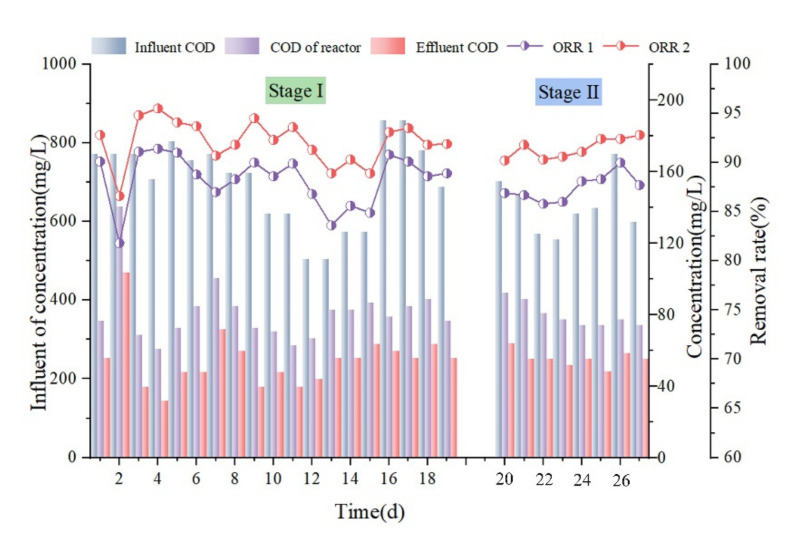
Chemical oxygen demand (COD) removal.

COD removal is shown in Figure 2. As mentioned in Section 1 and as presented in Figure 1, the oil production wastewater enters the reaction system and is first treated by aerobic microorganisms in the reactor. The water is then filtered out through a ceramic plate membrane to further reduce its COD. As shown in Figure 2, the COD concentration of the influent water ranged from 504 to 856.8 mg/L in the first stage. The effluent water COD fluctuated only during the first two days because the activated sludge was unable to adapt to the new environment, which necessitated the cultivation and restoration of its activity; the removal rate stabilized after the third day. The COD removal rate in the reactor of the aerobic microbial treatment alone reached more than 82.6%, while the rate achieved by ceramic plate membrane filtration was maintained at 88.7–95.2%, indicating good sludge activity during the test, wherein most organic pollutants were effectively removed during the reactor treatment. During the second stage of the test, the influent COD varied in the range of 554.4–772.8 mg/L, and the reactor COD was maintained below 87.36 mg/L. The final effluent COD was in a more stable range of 47.04–63.84 mg/L, and the COD removal rate was maintained at more than 90.6%. During this stage, the reaction system efficiently removed most organic matter in the feed water, maintaining stable and good effluent water quality.

#### 3.1.2. NH_4_^+^−N Removal

**Figure 3:**
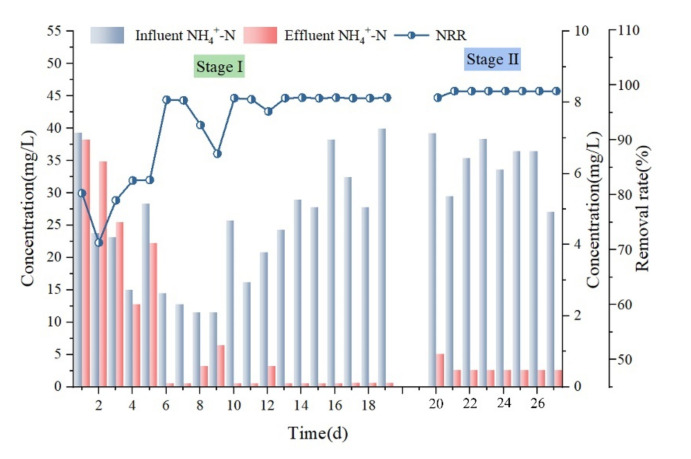
Ammonia nitrogen (NH_4_^+^−N) removal.

The NH_4_^+^−N removal effect is shown in Figure 3. As seen in Figure 3, the newly added activated sludge needs to be cultivated to recover its NH_4_^+^−N treatment capacity at the beginning of the first stage of the test process. The NH_4_^+^−N removal rate gradually increased from 71.6% to 83.8% for the first five days of reactor operation. Except for the sudden increase in NH_4_^+^−N load on day 9, when the influent NH_4_^+^−N increased from 11.5 mg/L to 25.7 mg/L, the removal rate fluctuated and was then maintained at 93.5~97.9% for the subsequent period, indicating that most influent NH_4_^+^−N was removed during C−MBR treatment after the sludge activity was restored by incubation. During the second stage, the effluent NH_4_^+^−N was maintained below 0.98 mg/L, and a stable removal rate of more than 97.5% and up to 98.9% was achieved. Subsequently, the removal rate was maintained at the highest value regardless of the variations in the inlet and outlet water, indicating that the aerobic microorganisms within the combined SBR and C−MBR process can efficiently treat NH_4_^+^−N.

**Figure 4:**
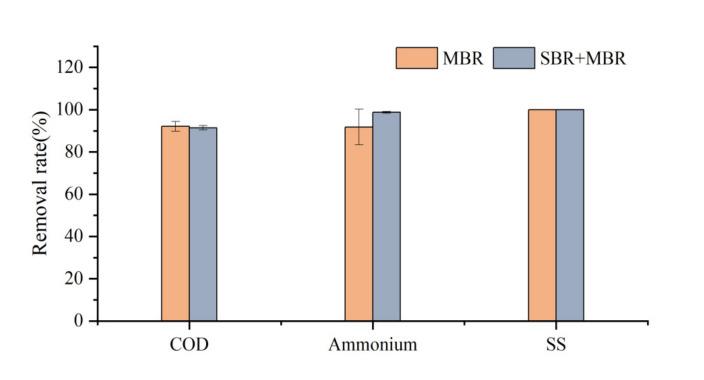
Comparison of removal rates of COD, NH_4_^+^−N, and SS between MBR and combined SBR and MBR processes.

**Figure 5:**
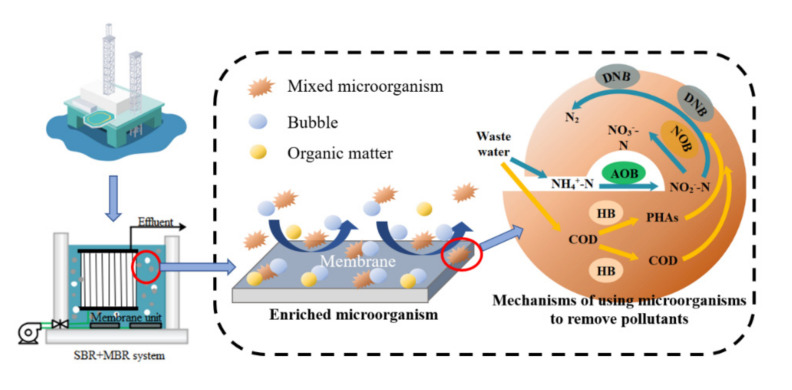
Microbial enrichment and removal mechanism of COD and NH_4_^+^−N in C−MBR reactor. (Mechanisms of using microorganisms to remove pollutants: The blue arrows indicate the denitrification process, including the conversion of NH_4_^+^−N to nitrate nitrogen (NO_3_^−^−N) under the action of ammonia oxidizing bacteria (AOB) and nitrite oxidizing bacteria (NOB) and the conversion of nitrite nitrogen (NO_2_^−^−N) to N_2_ under the action of denitrifying bacteria (DNB). Yellow arrows indicate the organic removal process. COD is degraded into small molecule COD by heterotrophic bacteria (HB) to facilitate the denitrification process.)

Overall, the effluent COD concentration after combined SBR and C-MBR treatment was 47.04–63.84 mg/L, with an average COD removal rate of more than 90%; the effluent NH_4_^+^−N concentration was below 0.98 mg/L, with an average NH_4_^+^−N removal rate of up to 98.8%. The effluent COD and NH_4_^+^−N satisfied the national comprehensive sewage discharge standard (GB8978-1996). Combining the two stages of COD and NH_4_^+^−N removal for comparison, the best removal was achieved at the beginning when combined with the SBR. The comparison of the removal rates of COD, NH_4_^+^−N, and suspended sludge (SS) by the MBR process alone and the combined SBR and C−MBR process is shown in Figure 4. Both processes achieved good COD and SS removal rates; the C−MBR process could completely remove SS, and the addition of SBR improved the nitrogen removal rate by 6.94%. The key to overcoming the treatment performance-related shortcomings of conventional biological processes is the effective retention of mixed microbial cells by process design and operation. Zhu et al. [20] compared the entrapped mixed microbial cell (EMMC) technology with encapsulated mixed microbial cells within the MBR process and observed that both the bilayer EMMC system and the single-stage MBR process showed efficient nitrogen removal performance, whereas the single-stage MBR process showed 10% higher COD removal than the bilayer EMMC system. Therefore, compared with other microbial enrichment technologies, the MBR process exhibited better treatment performance and considerably improved the effluent quality. However, industrial wastewater has a higher organic load than ordinary municipal wastewater, and the use of a single MBR process to treat this type of wastewater is energy intensive; therefore, a combined process can reduce the energy cost during process operation. For example, the average specific energy consumption of the combined conventional activated sludge (CAS) and MBR system is 0.6 kWh/m^3^ and can be as low as 0.76 kWh/m^3^ for the up-flow Anaerobic Sludge Bioreactor (UASB) and MBR process [19], while the specific energy consumption of the single-stage MBR process is 1.1 kWh/m^3^, which is higher than that of the combined processes [21]. Furthermore, SBR pre-treatment is necessary for the process. The C−MBR process certainly plays an important role in NH_4_^+^−N removal throughout the operation. This can be attributed to the enrichment of aerobic microorganisms in the C−MBR process, which effectively improves the nitrogen removal rate. The mechanism of mixed microbial enrichment and COD and NH_4_^+^−N degradation, migration, and transformation in the C-MBR system is shown in Figure 5.

In the correlation analysis listed in Table 2, there was an extremely strong positive correlation between COD concentration and organic removal rate (ORR) (*p* = 0.036 < 0.05) and an extremely strong negative correlation with nitrogen removal rate (NRR) (*p* = 0.04 < 0.05); there was an extremely strong positive correlation between C/N ratio and COD and ORR, an extremely strong negative correlation with NH_4_^+^−N (*p* = 0 < 0.01), and an extremely strong negative correlation with NRR. This phenomenon is the same as that described by Yadu et al. [22], where a high C/N ratio promotes COD removal, and under high C/N ratio conditions, heterotrophic-like bacteria become the dominant bacteria and the consumption of NH_4_^+^−N is naturally reduced. In the combined SBR and C−MBR process, the removal rate of ammonia nitrogen has been significantly improved. Combined with the analysis in Figure 5, at this stage, COD is more degraded into small molecule COD through heterotrophic bacteria (HB). Therefore, the ORR decreases and the NRR, which has a negative correlation with the ORR, shows a trend of increasing.

### 3.2. Microbial Diversity in the Reaction System

The community structures of A_0_ and B at the genus level are shown in Table 3. The dominant bacteria in A_0_ and B differed significantly, with Unclassified-Bacteria, Unclassified*-Bacteroidetes*, and Unclassified-Planctomycetaceae being dominant in A_0_ and *Marinobacterium*, *Marinobacter*, unclassified-Rhodobacteraceae, *Pseudidiomarina*, and *Thiomicrospira* being dominant in B. After technological enrichment, the genus *Marinobacterium* in sample B has a high abundance of 19.01%, which can synergistically degrade polycyclic aromatic hydrocarbons (PAHs) and effectively degrade petroleum substances in high-concentration oil extraction wastewater. They are major contributors to the removal of organic matter. The relative abundance of *Nitrosomonas* in A_0_ with ammonia oxidation was 0.17%, while the process enriched *Nitrosomonas* to a higher abundance of 7.04%. These results indicate that the NRR gradually increased with the enrichment of ammonia oxidizing bacteria (AOB) during the pilot run and stabilized after reaching a high removal rate of 98.9%. Due to the high C/N ratio of the high-salinity oil recovery wastewater in this study (C/N ratio range of 16.07 to 63.02), a higher proportion of HB such as *Marinobacterium* was allowed, reducing the abundance of *Nitrosomonas*, but to a lesser extent. The genus *Marinobacterium* and *Marinobacter* are species with specific functions for COD degradation, and through their activity, they facilitated the conversion of NO_2_^−^−N to N_2_ by Nitrosomonas, thus promoting the simultaneous removal of NH_4_^+^−N and COD. Therefore, the symbiosis between *Marinobacterium* and *Marinobacter* with *Nitrosomonas* as the dominant bacteria in this combined process and the enrichment effect of the membrane bioreactor simultaneously removed NH_4_^+^−N and COD with increased efficiency. In addition, high-salinity is a characteristic of marine oil recovery wastewater, which affects the bacterial community structure and threatens the MBR system’s treatment performance. Wang et al. [23] observed that a Cl^−^ concentration higher than 3.5% inhibits AOB growth, and 7% salinity causes the MBR system to collapse, which is irrecoverable. Therefore, the effect of salinity on microbial communities and system performance should be investigated when treating such wastewater.

### 3.3. Membrane Contamination

**Figure 6:**
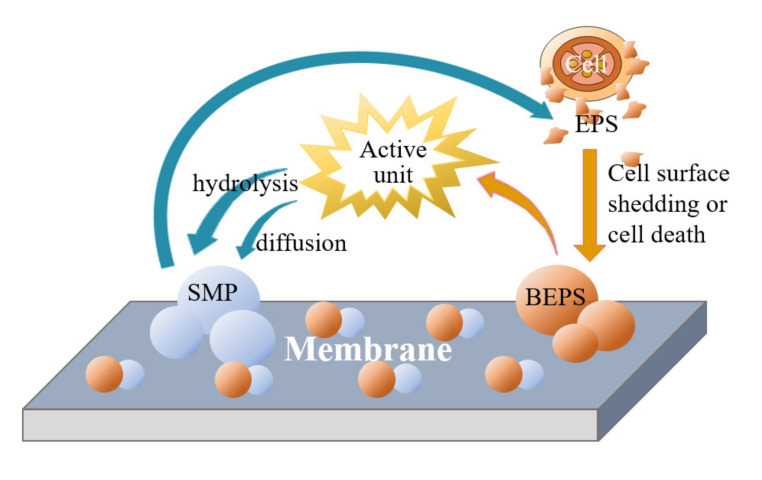
Mechanism of membrane fouling caused by EPS and SMP. (EPS: extracellular polymer; BEPS: fixed EPS; SMP: soluble microbial product; active unit: bacterial micelles and biofilm.).

Membrane contamination caused by extracellular polymer substances (EPSs) and soluble microbial products (SMPs) has received widespread attention and includes dissolved and insoluble macromolecular organic matter, colloidal particles, and solute molecules or particulates produced by microbial metabolism that clog membrane pores, thereby affecting the membrane flux [24,25,26,27]. The mechanism of membrane contamination caused by EPS and SMP is shown in Figure 6. The control of membrane fouling usually uses a backwash strategy, and NaClO solution has a significant cleaning effect on ceramic membranes. High-concentration NaClO is widely used in chemically enhanced backwashing (CEB), but it negatively affects membrane performance and microbial activity. Therefore, it is necessary to develop lower-concentration NaClO solutions to clean ceramic membranes. Yue X. et al. [28] obtained the optimal concentration through the comparative experiments of four NaClO solutions with different concentrations. Using 1 mg/L NaClO solution to clean the ceramic membrane can effectively alleviate the membrane fouling. According to the results of this study, a 1 mg/L NaClO solution was used for the backwashing of ceramic membranes in this study. Although the blower exhibits a certain flushing effect on the polluted flat membrane surface, the periodic backwashing by the control cabinet and solenoid valve also exerts a cleaning effect against the pollutants adsorbed onto the membrane pores. However, the membrane pressure tends to increase over time.

**Figure 7:**
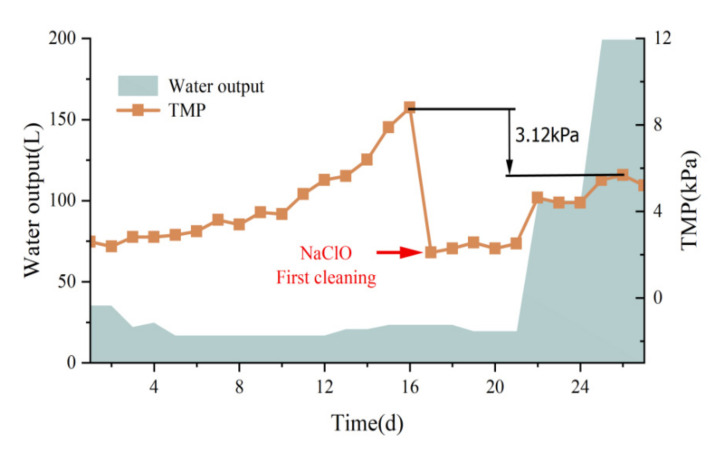
Changes in membrane pressure before and after cleaning.

**Figure 8:**
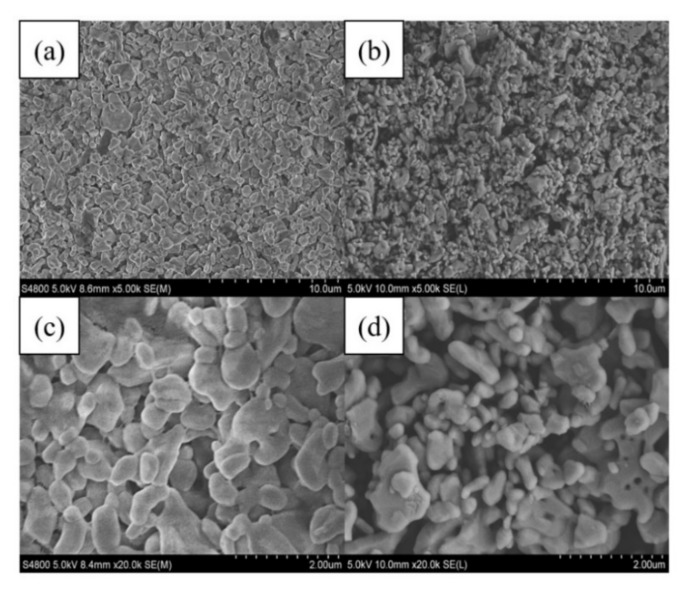
Scanning electron microscope (SEM) result of ceramic membrane. (**a**,**b**) are the 5000× magnified SEM images of the membrane before and after cleaning, respectively, and (**c**,**d**) are SEM images at 2000× magnification before and after membrane cleaning, respectively.

This pilot test formally operated for 27 d. Considering the increase in TMP, the process can be divided into two parts:(1) the first 16 d and (2) from day 17 to the end of operations. The membrane pressure changes were measured at different membrane output volumes (Figure 7). On the first day of the test, the TMP was 2.6 kPa, and the membrane pressure increased significantly over time, reaching 8.8 kPa on day 16, with a development rate of 0.39 kPa/d. On day 17, after cleaning with NaClO solution, the TMP rapidly decreased to 2.1 kPa, which is attributed to the strong oxidation property of NaClO solution that can oxidize the membrane surface and its internal pores. NaClO solution can oxidize the microscopic impurities inside the membrane pores, reduce the membrane pressure, and improve the membrane flux recovery [29]. The membrane effluent reached up to 200 L during the subsequent experiments, but the membrane pressure did not increase until the end of the experiment; a TMP of 5.2 kPa was achieved, and the development rate of TMP in the second stage was significantly reduced to 0.28 kPa/d. The results presented in Figure 7 show that the ceramic membrane surface can form a stable film layer, which continuously maintains the C−MBR reactor operation under low TMP conditions. Membrane contamination was effectively reduced, and the effect of cleaning the ceramic flat membrane once using NaClO solution was significant. In addition, the TMP development rate was lower compared to the study by Tien et al. [30], indicating that this combined process reduces the frequency of NaClO cleaning, thereby reducing the chemical cleaning cost. Scanning electron microscope (SEM) is the most direct method to examine the cleaning effect of NaClO solution, and Figure 8 shows the SEM images of the ceramic membranes before and after cleaning at 5000× and 20,000× magnification. In the cleaned membrane, the ceramic particles of which can be observed, the pore structures of the multilayer are formed by ceramic particles, and the irregular particles that are stacked to form pores are zigzag in shape and of various sizes compared with the contaminated membrane. Although the NaClO solution removes most contaminants, the surface differs from that of the new membrane, and the contaminants within the membrane pores are not completely eliminated.

### 3.4. Economic Analysis

At the end of the pilot plant test, the effluent COD, NH_4_^+^−N, SS, and oil concentrations were effectively reduced by the combined SBR and C-MBR process and were lower than 60 mg/L, 1 mg/L, 0 mg/L, and 0.5 mg/L, respectively, thereby satisfying the national comprehensive effluent discharge standard (GB8978−1996) Class I. Therefore, this combined process can be used to treat high-salinity oil recovery wastewater. The design flow rate considered was 200 m^3^/d. The economic analysis is expressed in two parts: capital expenditure and operating cost, which can be estimated by referring to the cost data of the same scale system and the average selling price in the market.

#### 3.4.1. Capital Expenditure (CAPEX)

The capital expenditure includes the engineering construction cost, pipe network cost, and non-engineering cost. The full-scale engineering application is based on the pilot scale; as this study was conducted on a pilot scale, the raw water collection device, chemical cleaning tank, and treated effluent collection device were not considered and included a total effective volume of 400 m^3^. Combined with the current exchange rate (1 USD = 6.36 CNY) to calculate the capital expenditure for the full-scale engineering application, as shown in Table 4, the total capital expenditure for the process was estimated to be 251,717 USD.

#### 3.4.2. Operating Expenses (OPEX)

**Figure 9:**
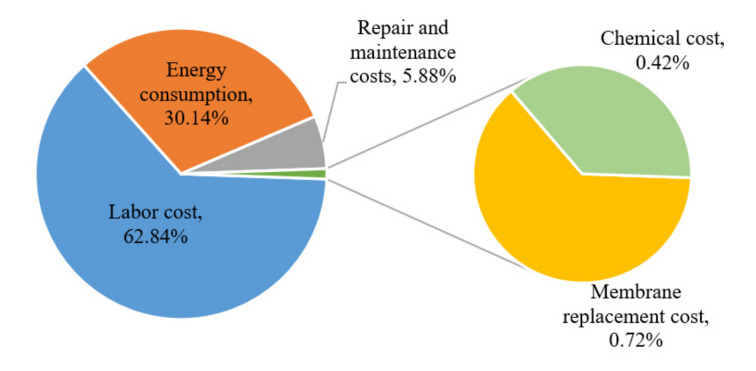
Percentage graph for each component of operating expenses.

Operating expenses include energy consumption, membrane replacement costs, maintenance and repair costs, chemical costs, and labor costs; the proportions of each component are shown in Figure 9. The energy consumption of this combined process is mainly from electricity, with an annual consumption of 41,391 kWh at an average unit price of 0.11 USD/kWh and an annual electricity cost of 4553.01 USD. Membrane replacement costs accounted for 2.4% of the energy consumption costs [31], which is approximately 109.27 USD/year. The maintenance and repair cost was 19.5% of the energy cost [31], which is approximately 887.84 USD/year. Depending on the treatment process requirements, the required chemicals include only NaClO, with an annual chemical cost of 63.28 USD. A worker with an annual salary of 9492.32 USD can operate and manage the plant due to the automatic control operation mode of the entire process. The daily wastewater treatment capacity was 200 m^3,^ and the unit wastewater treatment cost was 0.21 USD/m^3^.

The capital expenditure for operating the full-scale combined SBR and C−MBR process was 251,717 USD, and the cost for treating high-salinity oil recovery wastewater was 0.21 USD/m^3^. The energy cost of the conventional MBR process was 0.56 USD/m^3^, and the combined process reduced the energy cost by 62.5% [32].

## 4. Conclusions

The combined process of SBR and C−MBR effectively treated high-salinity oil recovery wastewater, and the pollutant indexes COD and NH_4_^+^−N were reduced to less than 60 mg/L and 0.98 mg/L, respectively, with the removal rate reaching 93% and 98.9%, respectively. MBR enrichment is the key to high processing performance. The symbiosis between *Marinobacterium*, *Marinobacter*, and *Nitrosomonas* as the dominant bacterial species in this combined process contributed to the simultaneous removal of NH_4_^+^−N and COD with increased efficiency, and the abundance of *Nitrosomonas* enriched in the reactor was 6.87% higher than that in the domesticated culture of the municipal wastewater treatment plant, which significantly improved the nitrogen removal rate. The enrichment effect of MBR increases the susceptibility of ceramic membranes to membrane contamination; using NaClO solution can considerably remove membrane contaminants and clean ceramic membranes, which can reduce the cleaning frequency of ceramic membranes and the chemical cost. Finally, based on the pilot application study, the capital expenditure for operating the full-scale combined SBR and C−MBR process was 251,717 USD, and the unit wastewater treatment cost was estimated to be 0.21 USD/m^3^, which saves 62.5% of the energy cost compared with the traditional MBR process.

## Figures and Tables

**Table 1:** Quality of oil production wastewater.

Parameter	Unit	Influent
Temperature	°C	38–50
pH	-	7.1–8.2
Total salinity	g/L	27.4–31.8
SS	mg/L	140–610
COD	mg/L	100–1479
BOD_5_	mg/L	36.1–650
NH_4_^+^−N	mg/L	11.3–40.2
TN	mg/L	9–13
TP	mg/L	7–12
Oil	mg/L	11.5–15

**Table 2:** Pearson correlation analysis.

	COD	NH_4_^+^−N	ORR	NRR
NH_4_^+^−N	−0.014			
ORR	0.406 *	−0.214		
NRR	−0.398 *	0.248	−0.107	
C/N ratio	0.357	−0.880 **	0.336	−0.286

* Correlation significant at 0.05 (two−tailed). ** Correlation significant at 0.01 (two−tailed).

**Table 3:** Community structure at the genus level of sludge samples.

Sample	Genus	Contain (%)	Sample	Genus	Contain (%)
A_0_	Unclassified-Bacteria	16.68	B	*Marinobacterium*	19.01
	Unclassified-*Bacteroidetes*	8.04		*Marinobacter*	17.61
	Unclassified-Planctomycetaceae	7.44		Unclassified-Rhodobacteraceae	16.2
	*Nitrospira*	1.22		*Pseudidiomarina*	14.08
	*Gp10*	0.68		*Thiomicrospira*	11.27
	Unclassified-Rhodobacteraceae	0.18		*Meyerozyma*	9.59
	*Nitrosomonas*	0.17		*Nitrosomonas*	7.04
	*Mycobacterium*	0.02		*Mycobacterium*	2.11
	Unclassified-*Rhodospirillaceae*	0.02		Unclassified-*Rhodospirillaceae*	2.11

**Table 4:** C−MBR combined process engineering application capital expenditure description table.

Project		Quantity	Unit	Unit Price (USD)	Total Price (USD)	Remark
Construction cost	Raw pool, SBR pool, MBR pool, chemical cleaning medicine tank, water outlet pool	1	Item	/	56,600	The tank is built with a reinforced concrete structure, with a thickness of about 300 mm
	Subtotal	/	/	/	56,600	
Non-engineering costs	PLC control system	1	Set	/	157,000	The total power of the PLC control cabinet is 3.5 kw
	Lift pump	4	Tower	239	960	2 use 2 backup; the power is 0.25 kw
	High-precision electromagnetic flowmeter	2	Set	157.50	315	
	Ceramic membrane module	148	Piece	78.60	11,700	148 pieces per group (including interface, silicone tube, etc.)
	Microfiltration membrane self-priming pump	2	Tower	158.50	317	1 use and 1 backup; the power is 0.45 kw
	Cleaning system	1	Set	/	3160	With CIP pump, the power of the pump is 2.2 kw
	Aeration system	2	Set	315	630	Including pipeline valve
	Aeration blower	4	Tower	395	1580	2 use 2 backup; the power is 0.55 kw
	Shipping fee	1	Item	/	2390	
	Commissioning fee	1	Item	/	3440	
	Subtotal	/	/	/	181,492	
Pipe network cost	Pipe network	1	Set	/	9500	Including pipes, fittings, etc.
	Wire and cable	1	Set	/	1100	
	Hardware parts	1	Set	/	745	
	Plumbing installation fee	1	Item	/	2280	
	Subtotal	/	/	/	13,625	
Total		/	/	/	251,717	

## Data Availability

The datasets analyzed during the current study are available from the corresponding author on reasonable request.
